# Exploration of Key Regulatory Factors in Mesenchymal Stem Cell Continuous Osteogenic Differentiation via Transcriptomic Analysis

**DOI:** 10.3390/genes15121568

**Published:** 2024-12-04

**Authors:** Yu Pan, Tao Liu, Linfeng Li, Liang He, Shu Pan, Yuwei Liu

**Affiliations:** 1 Department of Orthopedic Surgery, The Affiliated People’s Hospital of Jiangsu University, Zhenjiang 212002, China; panyu@stmail.ujs.edu.cn (Y.P.); taotao4426@aliyun.com (T.L.); 2 School of Medicine, Jiangsu University, Zhenjiang 2012013, China; 3 Department of Orthopedic Surgery, Southwest Hospital Jiangbei Area (The 958th Hospital of Chinese People’s Liberation Army), Chongqing 400020, China; lilinfeng0809@163.com; 4 School of Medicine, Tongji University, Shanghai 201619, China; heliangasd123@163.com; 5 Computer Science School, Jiangsu University of Science and Technology, Zhenjiang 212003, China

**Keywords:** MSCs, lineage change, transcriptomic sequencing, dynamic regulation, osteogenesis

## Abstract

Background/Objectives: Mesenchymal stem cells (MSCs) possess the remarkable ability to differentiate into various cell types, including osteoblasts. Understanding the molecular mechanisms governing MSC osteogenic differentiation is crucial for advancing clinical applications and our comprehension of complex disease processes. However, the key biological molecules regulating this process remain incompletely understood. Methods: In this study, we conducted systematic re-analyses of published high-throughput transcriptomic datasets to identify and validate key biological molecules that dynamically regulate MSC osteogenic differentiation. Our approach involved a comprehensive analysis of gene expression patterns across human tissues, followed by the rigorous experimental validation of the identified candidates. Results: Through integrated analytical and experimental approaches, we utilized high-throughput transcriptomics to identify four critical regulators of MSC osteogenic differentiation: *PTBP1*, *H2AFZ*, *BCL6*, and *TTPAL (C20ORF121)*. Among these, *PTBP1* and *H2AFZ* functioned as positive regulators, while *BCL6* and *TTPAL* acted as negative regulators in osteogenesis. The regulatory roles of these genes in osteogenesis were further validated via overexpression experiments. Conclusions: Our findings advance our understanding of MSC differentiation fate determination and open new therapeutic possibilities for bone-related disorders. The identification of these regulators provides a foundation for developing targeted interventions in regenerative medicine.

## 1. Introduction

Mesenchymal stem cells (MSCs) possess the ability to differentiate into diverse cell lineages [1,2], rendering them indispensable for maintaining physiological homeostasis, as well as promoting tissue regeneration and repair [3,4]. MSCs are ubiquitously distributed across multiple tissues and constitute a multipotent progenitor cell population with clonogenic potential [5,6]. Consequently, MSCs have garnered significant attention owing to their immense therapeutic potential in regenerative medicine [7,8]. The fate determination of MSCs is tightly regulated by intricate interactions among numerous cell factors derived from the tissue microenvironment [9], which act as molecular switches for lineage differentiation through specific activation or dysfunction mechanisms [10,11]. Understanding the molecular mechanisms that determine the MSC fate is essential for implementing targeted strategies to correct abnormal lineage distribution, particularly in conditions such as osteoporosis and bone aging [12,13].

Previous studies have identified several cellular factors, such as Runx2 [14,15] and Osterix [16,17], as the key regulators of MSC differentiation, particularly in promoting osteogenic lineage differentiation [18,19,20]. Genomics has furthered research conducted in the field of genome-wide dynamics of transcription factor binding and epigenome programming during preosteoblast differentiation [21,22]. However, further research is required to elucidate the dynamics of chromosome structure and enhancer activity during osteogenesis in MSC lineage studies [23,24]. To date, no studies have systematically explored the cellular factors that continuously regulate MSC differentiation into the osteogenic lineage across different time points, hindering a comprehensive evaluation of osteoporosis [25,26]. Therefore, it is crucial to conduct systematic investigations into the cellular factors governing MSC lineage fate to identify key elements that influence MSC differentiation [27,28].

In this study, we performed a systematic analysis of differentially expressed genes (DEGs) by integrating high-throughput sequencing data related to human MSC differentiation into the osteogenic lineage [29]. Subsequently, we identified DEGs with significant differential expression across multiple time points and validated the candidate genes exhibiting high expression in bone marrow tissue under screening conditions by comparing them to 45 human tissues from the HUMAN PROTEIN ATLAS database. Finally, we performed biological experiments to verify the ability of the selected candidates to continuously regulate the osteogenic induction of MSCs. Our study established a foundational framework for identifying candidates with enhanced clinical relevance for the treatment of osteoporosis and bone fracture healing.

## 2. Materials and Methods

### 2.1. Cell Culture

Primary Mouse bone marrow mesenchymal stem cells (mBMSCs) were isolated from the femur bone marrow of 2-month-old wild-type C57BL/6J mice. The isolated cells were stored in a BMSC medium (DMEM medium containing 20% heat-inactivated FBS) for 1 day. On the second day, the supernatant (including osteoclasts) was removed, and the adherent hematopoietic cells were removed by intensive washing with phosphate-buffered saline (PBS) three times. The culture was continued with a fresh BMSC medium. After 2 weeks, the growth colonies were collected by trypsinization for further passage and differentiation. All the experimental and control group mice were derived from the same cage. Each replicate was derived from a different mouse. The experiments were conducted in triplicate.

Mycoplasma screening was performed on the BMSC cell line using the highly sensitive Mycoplasma PCR Detection Kit (Catalog# K0103, HUABIO, Hangzhou, China). The results confirmed the absence of mycoplasma contamination, thereby ensuring the integrity and reliability of our experimental system.

### 2.2. Flow Cytometry Analysis

Passage four mBMSCs isolated from the C57BL/6J mice were characterized by flow cytometry analysis. A total of 1 × 10^6^ cells were washed with 10% FBS/PBS and centrifuged at 1000 rpm for 5 min. The cell pellets were incubated at 4 °C with FITC-conjugated mice anti-CD90, CD73, CD105, CD34, and CD117. The cells were examined by flow cytometry with 10,000 events recorded for each condition, and data were analyzed using FlowJo (v10.8.1).

### 2.3. Cell Differentiation

BMSCs were cultured in α-modified Eagle’s minimum essential medium supplemented with 10% FBS, 200 mM L-glutamine (25030081, Gibco, Thermo Fisher, Waltham, MA, USA), and nonessential amino acids (NEAA, 11140050, Gibco).

Osteogenic differentiation: 100 mM ascorbic acid, 2 mM b-glycerophosphate, and 10 nM dexamethasone (D4902; Sigma–Aldrich, Merck KGaA, Darmstadt, Germany).

For adipogenic differentiation, 0.5 mM isobutylmethylxanthine (IBMX, HY-12318, MedChemExpress, Monmouth Junction, NJ, USA), 0.5 mM hydrocortisone (803146, Sigma–Aldrich, Germany), and 60 mM indomethacin (I7378, Sigma–Aldrich, Germany) were used.

Media were changed every 2 days.

### 2.4. Lentivirus Transduction

Lentiviral transduction: Lentivirus-overexpressing genes were purchased from Genomeditech (Shanghai, China).

BMSCs were infected with the virus. After 48 h transfection, the cells underwent osteogenic-induced differentiation.

### 2.5. Quantitative Real-Time PCR

Total RNA from the cultured cells was isolated during TRlzolTM reagent (lnvitrogenTM, Thermo Fisher, Waltham, MA, USA) following the manufacturer’s protocol [30,31]. Subsequently, cDNA synthesis was performed using the PrimeScriptTM RT Reagent Kit (cat. no. RR0114A, Takara Bio Inc., Kusatsu, Japan) according to the manufacturer’s instructions. Finally, a qPCR analysis was conducted on a LightCycler^®^ 96 system (Roche, Basel, Switzerland). The experiment was repeated three times. All the data were normalized to those of GAPDH. The relative expression levels of the target genes were calculated using the 2^−ΔΔCT^ method. All the data are presented as means ± SD from three independent experiments [32]. The sequences of the primers used are listed in Table S1.

### 2.6. Alizarin Red Staining (ARS)

The cell-abandoned medium was immersed in alizarin red S staining solution for 30 min. The cells were quickly rinsed with distilled water and then studied under a microscope.

### 2.7. Alcian Blue Staining

The cells were washed with PBS three times for 3 min each and then fixed with 4% paraformaldehyde. Next, the cells were washed with PBS again and incubated with 1% of alcian blue for 30 min. Then, the cells were rinsed in water for 2 min and dehydrated with 95% ethanol for 15 s. Ultimately, the slides were observed under a microscope.

### 2.8. Oil Red O Staining

The cells were immobilized with phosphate buffer containing 10% formaldehyde for 10 min and then rinsed once with PBS for 1 min. Next, the cells were rinsed with 60% isopropyl alcohol for 15 s to promote neutral fat staining. Then, the cells were stained with filtered oil red O working solution at 37 °C for 30 min, treated with 60% isopropyl alcohol for 30 s, and rinsed with PBS three times for 3 min each. Finally, the cells were observed under a microscope.

### 2.9. ALP Staining

After 7 days of osteogenic induction, the cells were washed with PBS three times and then fixed with 4% paraformaldehyde. Subsequently, the cells were incubated with a solution containing 5-bromo-4-chloro-3-indolyl phosphate/nitro blue tetrazolium. After 15 min of incubation at 37 °C, the cell layer was washed with deionized water three times and observed under a digital camera.

### 2.10. Bioinformatics Analysis

#### 2.10.1. Data Collection and Processing

Microarray datasets in this study were obtained from the Gene Expression Omnibus (GEO) database. Datasets were included based on the following criteria: (1) the high-throughput sequencing or array-based transcriptome analysis of MSC osteogenic differentiation; (2) total sample size greater than 15; and (3) the availability of raw data with clear experimental annotations. Two datasets meeting these criteria were selected: GSE37558 (12-day osteogenic induction) and GSE28205 (14-day osteogenic induction) [33,34].

#### 2.10.2. Differential Expression Analysis

Raw data preprocessing and normalization were performed using the R/Bioconductor package. A differential expression analysis was conducted using GEO2R with the following parameters: false discovery rate (FDR) ≤ 0.25 and *p*-value ≤ 0.05. Heatmaps were generated to illustrate the differences between the groups in each dataset. Venn diagram analysis was performed using the VennDiagram R package, and the overlapping differentially expressed genes (DEGs) in the two datasets were identified as common DEGs (co-DEGs). Further refinement of the co-DEGs was achieved by applying an additional threshold of log2(fold change) ≥ 1.5. The final set of DEGs was categorized into up- and down-regulated genes based on their expression patterns for subsequent experimental validation.

To assess the tissue-specific expression profiles of the identified DEGs, we utilized the Human Protein Atlas database [35,36,37]. The expression levels of the candidate genes were systematically evaluated in osteogenic-relevant tissues, specifically bone marrow. Genes exhibiting elevated expression in these tissues, as defined by standardized database parameters, were prioritized for further experimental validation. This tissue-specific filtering approach enabled the identification of DEGs with potentially relevant biological functions in osteogenic contexts.

## 3. Results

### 3.1. Integration Analysis of Microarray Datasets to Identify Differentially Expressed Genes in MSC Osteogenic Differentiation

To identify the genes associated with MSC osteogenic differentiation, we performed an integration analysis on two microarray datasets with comparable levels of osteogenic induction. Specifically, the datasets utilized for our analysis were GSE37558 for 12-day osteogenic induction and GSE28205 for 14-day induction. Despite the difference in induction duration, both datasets were considered to have comparable levels of osteogenic activity, making them suitable for a combined analysis.

**Figure 1:**
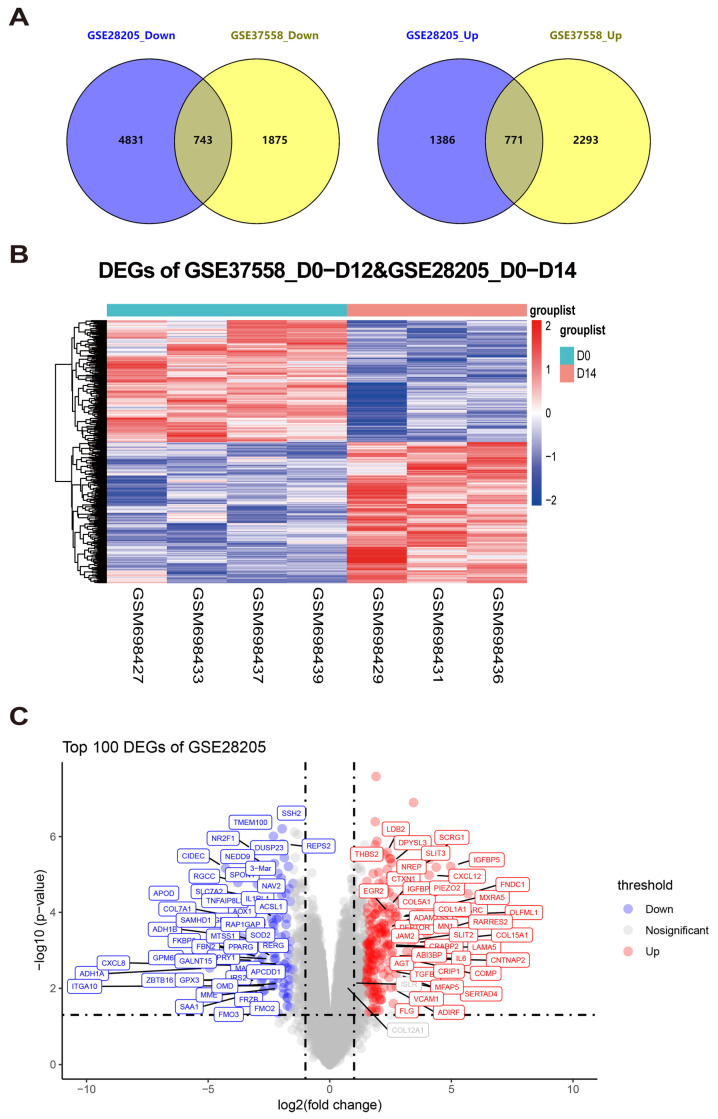
Two batches of microarray datasets related to long-term osteogenic induction were analyzed to identify DEGs. (**A**) Venn diagrams illustrating the intersection of differentially expressed genes (DEGs) between the two microarray datasets. The left panel represents the down-regulated genes, while the right panel represents the up-regulated genes in GSE28205 and GSE37558. The DEGs were identified using thresholds of false discovery rate (FDR) ≤ 0.25 and *p*-value ≤ 0.05. (**B**) A heatmap was utilized to visualize the DEGs between the two batches of datasets. (**C**) The volcano plot in panel C presents the information on the DEGs with a cutoff value of log2(fold change) ≥ 1.5.

We employed a flexible threshold with a false discovery rate (FDR) ≤ 0.25 and a *p*-value ≤ 0.05 to detect DEGs within each dataset. Moreover, a Venn diagram analysis was used to recognize the common DEGs between the two datasets, revealing a total of 1156 shared genes (Figure 1A,B).

To further refine the selection, we applied a cutoff of log2(fold change) ≥ 1.5, which identified 169 DEGs. Among these, 100 were down-regulated, and 69 were up-regulated (Figure 1C). The above genes would be subjected to further experimental validation and analysis for a deeper understanding of their roles in MSC osteogenic differentiation.

### 3.2. Exploration of Continuously Differentially Expressed Genes During MSC Osteogenic Induction from GSE37558 Dataset

To ensure a comprehensive analysis of transcriptome dynamics during osteogenic differentiation, we integrated microarray data from the GSE37558 dataset over a time course. The dataset enabled us to examine changes in gene expression across multiple time points during the differentiation process.

**Figure 2:**
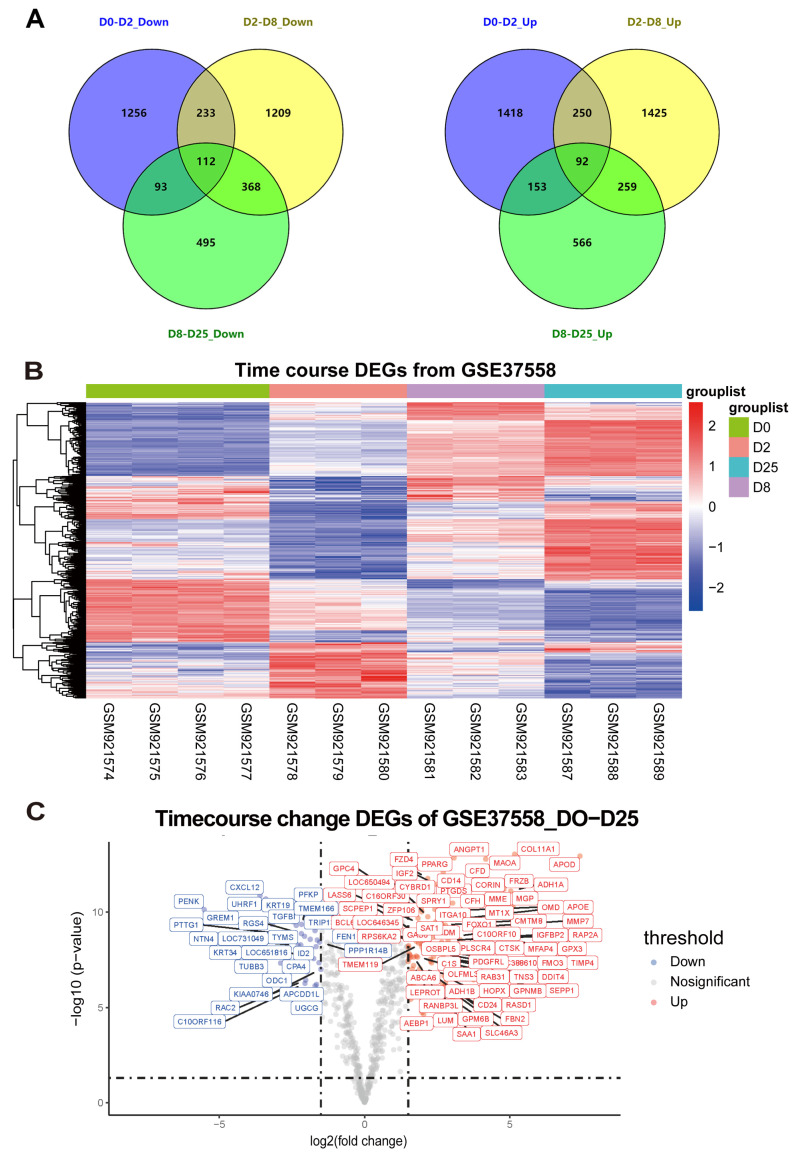
Refinement of time-dependent osteogenic induction in MSCs through the analysis of microarray dataset GSE37558. (**A**) A Venn diagram was generated to illustrate the overlap of DEGs across three time points during osteogenic induction in the GSE37558 dataset. The left panel represents the down-regulated genes, while the right panel represents the up-regulated genes. The DEGs were identified based on thresholds of false discovery rate (FDR) ≤ 0.25 and *p*-value ≤ 0.05. (**B**) The DEGs among the three different osteogenic induction time points in the GSE37558 dataset were visualized using a heatmap. (**C**) The volcano plot in panel C presents the information on the DEGs with a cutoff value of log2(fold change) ≥ 1.5.

For our analysis, we performed time points of osteogenic induction as a reference for the subsequent induction time points and analyzed the GSE37558 dataset. Specifically, we compared gene expression changes between Day 0 to 2, Day 2 to 8, and Day 8 to 25. To identify DEGs across these intervals, we applied screening criteria with a false discovery rate (FDR) of ≤0.25 and a *p*-value of ≤0.05. This approach allowed us to detect 549 DEGs over the course of osteogenic induction(Figure 2A,B).

Delving into our findings, we used a log2(fold change) threshold of ≥1.5, identifying 121 DEGs. Of these, 84 were up-regulated, and 27 were down-regulated (Figure 2C). These genes would undergo additional scientific investigation to further explore their roles in MSC osteogenic differentiation.

### 3.3. Integrated Time-Course Analysis of Differentially Expressed Genes During MSC Osteogenic Induction

We conducted an integrated time-course analysis of DEGs both with a single batch of microarray data and across multiple batches from different research groups. The approach optimized the identification of continuously differentially expressed genes during MSC osteogenic induction.

**Figure 3:**
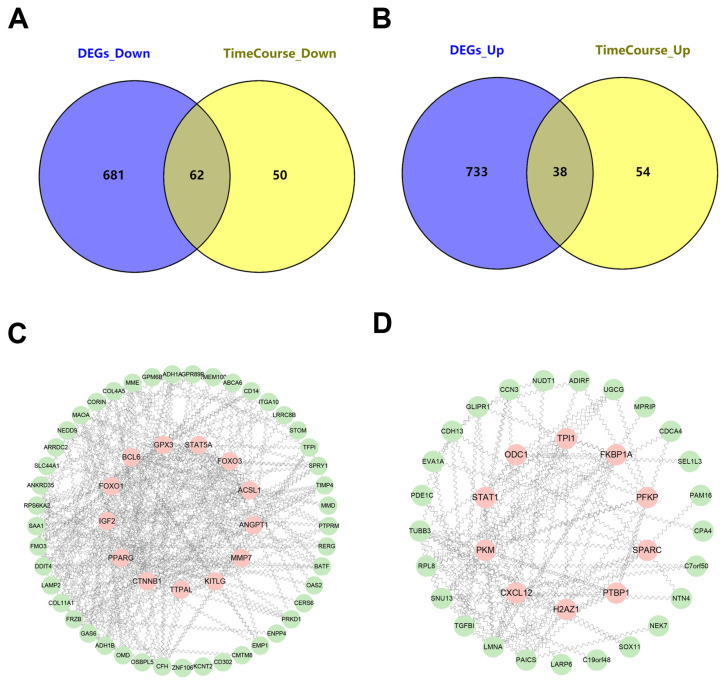
Recombining DEGs to enhance the screening and identification of key regulatory molecules. (**A**,**B**) Venn diagram showing the intersection of DEGs across time points and microarray datasets from various batches; (**C**,**D**) Detailed view of the coexpression network of DEGs, highlighting key genes.

We subsequently applied a log2(fold change) (LOGFC) threshold of ≥1.5, identifying 62 genes that were consistently down-regulated and 38 genes that were consistently up-regulated throughout the osteogenic induction process (Figure 3A,B).

To further investigate the regulatory role of these DEGs, we constructed coexpression networks to highlight potential key regulatory molecules in the STRING database (https://cn.string-db.org/), accessed on 24 November 2024.(Figure 3C,D). The network analysis revealed several hub genes, which exhibited strong coexpression with other DEGs. The results mentioned above suggest that the 49 up-regulated genes may play a crucial role in regulating the dynamics of MSC osteogenic differentiation. Follow-up investigations are currently underway to validate these candidate genes and explore their biological functions in the osteogenic process.

### 3.4. Identification of Key Regulators Governing MSC Osteogenic Differentiation Through the HUMAN PROTEIN ATLAS Database

We focused on identifying key regulators that influence the dynamic progression of MSC osteogenic differentiation. To achieve this, we integrated the expression levels of candidate genes in human tissues using the HUMAN PROTEIN ATLAS database, which provides comprehensive data on RNA and protein expression across 45 human tissue types.

The candidate genes, previously identified as potential regulators of MSC osteogenic differentiation, were analyzed for their expression in human tissues. We leveraged the HUMAN PROTEIN ATLAS database to assess their expression levels in both RNA and protein forms, allowing us to determine the relevance of these genes in various tissues.

Previous studies have highlighted the pivotal role of bone cells, including mesenchymal stem cells and hematopoietic stem cells, in regulating cellular behavior and maintaining tissue homeostasis within the stem cell lineage [38,39]. The skeletal system contains intricate cell lineages derived from these stem cells, which dictate their differentiation into osteogenic lineage, coupled with maintaining the homeostasis of both skeletal and marrow tissues [40].

Given the importance of hematopoietic tissues in blood and immune system regulation, we examined the expression of the candidate genes in these specific tissues using data from the HUMAN PROTEIN ATLAS. A total of 13 potential key regulators were identified, all of which exhibited high expression in some or all of these tissues. Detailed information is provided in Table 1.

Among the 13 candidates, four genes—*PTBP1*, *H2AFZ*, *BCL6*, and *TTPAL (C20ORF121)*—were found to have particularly high expression levels in most tissues related to the blood and immune system. Three of these genes were highly expressed across all four tissues, while one showed medium expression. The four genes, *PTBP1*, *H2AFZ*, *TTPAL*, and *BCL6*, serve as critical regulators of MSC osteogenic differentiation. Their potential roles in controlling this dynamic process make them promising targets for further investigation. We are currently conducting biological experiments to validate their molecular functions and further elucidate their involvement in MSC differentiation.

### 3.5. Isolation of Bone Mesenchymal Stem Cells and qRT–PCR Identification of Candidate Genes During Osteogenic Induction

mBMSCs are widely used in cell therapy and tissue engineering due to their self-renewal capacity and ability to differentiate into various mesoblastic cell types, including osteoblasts, chondrocytes, and adipocytes [41,42]. They are a type of multilineage progenitor cell that possesses self-renewal capacity and can differentiate into various types of mesoblastic cells, including osteoblasts, chondrocytes, adipocytes, etc. [43]. Given their significance, we isolated BMSCs to validate potential candidate genes identified through bioinformatics analysis.

**Figure 4:**
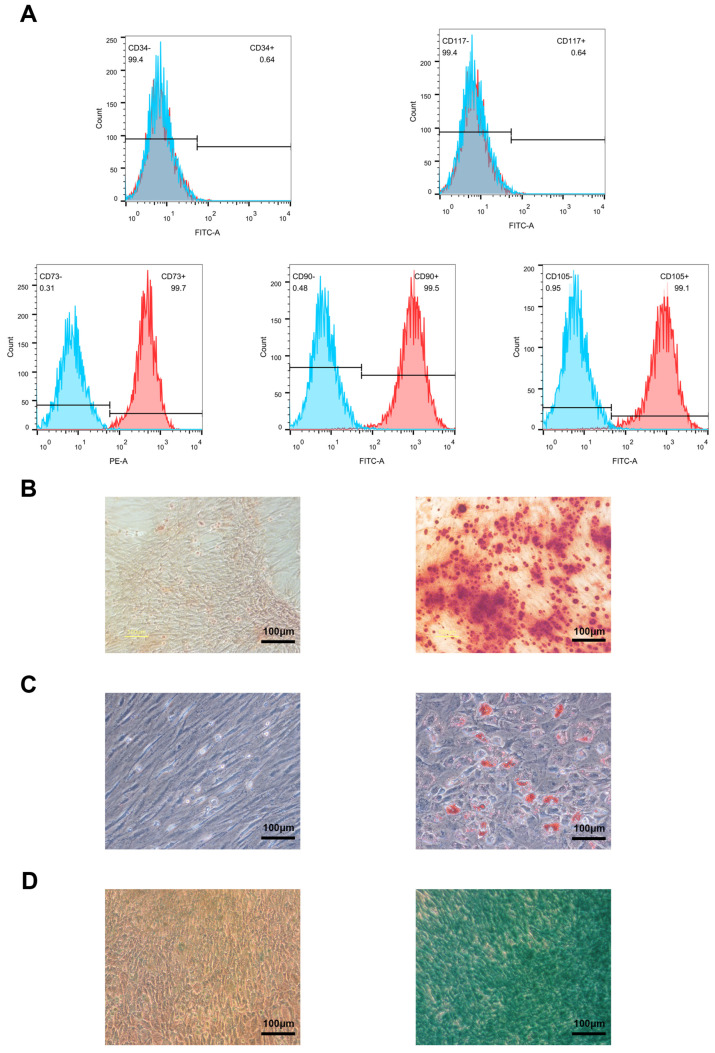
Characterization and multipotent differentiation capacity of mouse bone marrow mesenchymal stem cells (mBMSCs). (**A**) The flow cytometry analysis of the expression of positive (CD90, CD73, and CD105) and negative (CD34 and CD117) markers of mBMSCs. (**B**) Alizarin Red S staining for mBMSCs culturing for 21 days in osteogenic medium or basal medium. (**C**) Oil Red O staining for mBMSCs culturing for 14 days in adipogenic medium or basal medium. (**D**) Alcian blue staining for mBMSCs culturing for 28 days in chondrogenic medium or basal medium with the method of micromass. Scale bar: 100 μm. All the data are presented as the means ± SEMs. Statistical significance was determined using one-way ANOVA followed by Scheffe’s post hoc test. All the cell experiments were repeated independently in triplicate.

The isolated mBMSCs expressed high levels of mesenchymal markers CD90 (99.5%), CD73 (99.7%), and CD105 (99.1%), while lacking hematopoietic and myeloid markers CD34 (0.64%) and CD117 (0.64%), as confirmed by flow cytometry (Figure 4A). Their multilineage differentiation potential was demonstrated through osteogenic, adipogenic, and chondrogenic induction, evidenced by Alizarin Red, Oil Red O, and Alcian Blue staining, respectively (Figure 4B–D).

**Figure 5:**
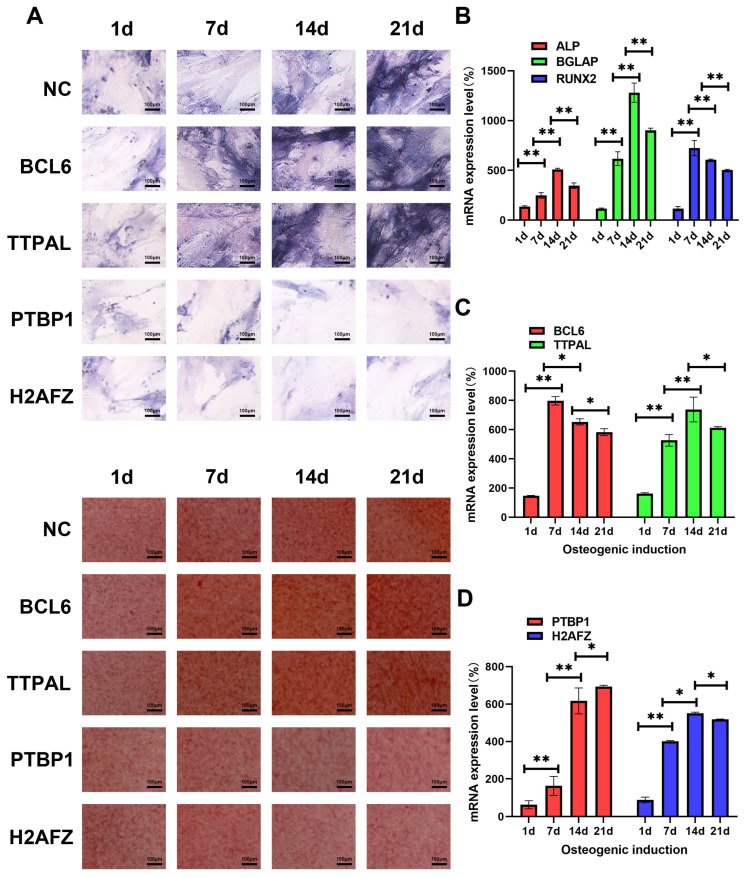
Elucidating the molecular mechanisms governing dynamic osteogenic induction in MSCs through the overexpression of four candidate genes using lentiviral packaging technology. (**A**) ALP and ARS were performed to evaluate the osteogenic differentiation of BMSCs at 1, 7, 14, and 21 days. Scale bar: 100 μm. (**B**) A qRT–PCR analysis was conducted to assess the mRNA expression levels of osteogenic markers (*ALP*, *BGLAP*, and *RUNX2*) at 1, 7, 14, and 21 days. (**B**) A qRT–PCR analysis was conducted to assess the mRNA expression levels of osteogenic markers (*ALP*, *BGLAP*, and *RUNX2*) at 1, 7, 14, and 21 days. (**C**) The temporal expression of *BCL6* and *TTPAL* mRNA during the osteogenic induction period was analyzed at 1, 7, 14, and 21 days via qRT–PCR. (**D**) The temporal expression of *PTBP1* and *H2AFZ* mRNA during the osteogenic induction period was analyzed at 1, 7, 14, and 21 days via qRT–PCR. The experiments were performed in triplicate using cells isolated from three separate 2-month-old wild-type C57BL/6J mice. Each replicate was derived from a different mouse. All the data are presented as the means ± SEMs. Statistical significance was determined using one-way ANOVA followed by Scheffe’s post hoc test. * *p* < 0.05; ** *p* < 0.01. All the cell experiments were repeated independently in triplicate.

We isolated mBMSCs and confirmed their osteogenic differentiation potential through ALP and ARS assays (Figure 5A).

For osteogenic differentiation, BMSCs were treated with an osteogenic medium, and RNA was collected on Day 1, Day 7, Day 14, and Day 21. To assess osteogenesis, we measured the expression of osteogenesis, including ALP, BGLAP, and RUNX2 (Figure 5B).

Next, we evaluated the expression of four candidate genes—*BCL6*, *TTPAL*, *PTBP1*, and *H2AFZ*. Using qRT-PCR, we assessed their RNA expression on Day 1, Day 7, Day 14, and Day 21. *BCL6* and *TTPAL* showed high expression throughout the induction (Figure 5C). In contrast, *PTBP1* and *H2AFZ* exhibited a decrease in expression over time (Figure 5D).

### 3.6. Identifying the Molecular Function of Osteogenic Regulators in MSCs via Lentiviral Overexpression of Candidate Genes

To explore the molecular mechanisms that regulate osteogenic induction in MSCs, we performed lentiviral packaging technology to overexpress four potential regulatory genes: *PTBP1*, *H2AFZ*, *BCL6*, and *TTPAL*.

**Figure 6:**
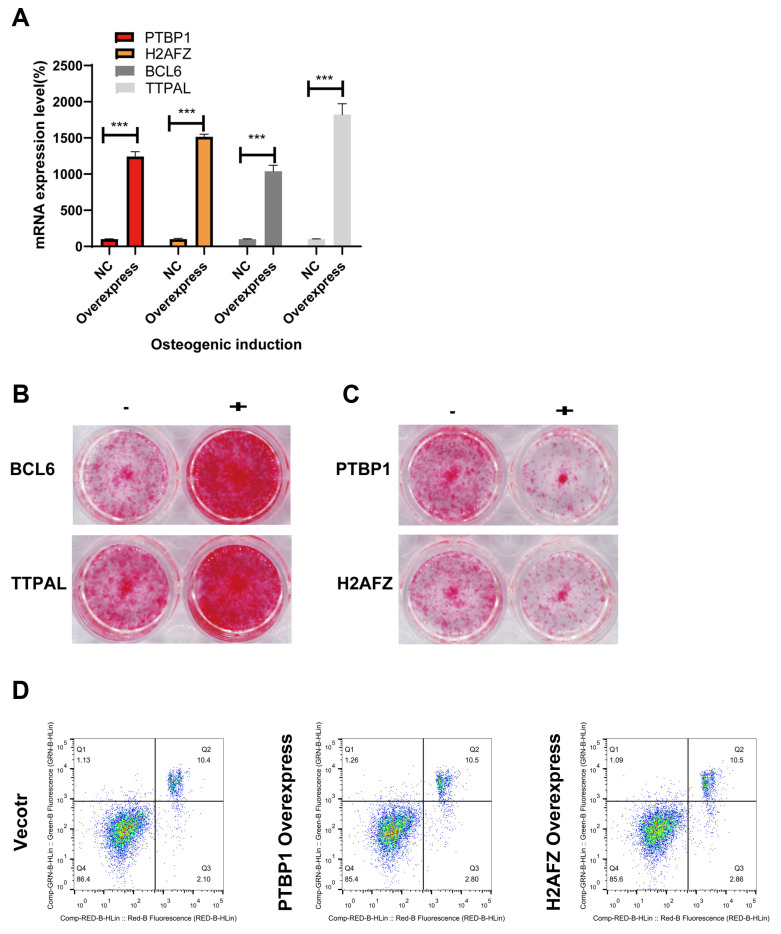
Molecular functions involved in the dynamic regulation of osteogenic induction through the lentiviral-mediated overexpression of four candidate genes. (**A**) Overexpression efficacy measured by qRT–PCR; (**B**) the overexpression of *BCL6* and *TTPAL* affects the osteogenic phenotype of BMSCs, as detected by an ARS assay; (**C**) the overexpression of *PTBP1* and *H2AFZ* alters the osteogenic phenotype of BMSCs, as detected by an ARS assay; (**D**) flow cytometry analysis showing the effects of *PTBP1* and *H2AFZ* overexpression on apoptosis. Each replicate was derived from a different mouse. All the data are presented as the means ± SEMs. Statistical significance was determined using one-way ANOVA followed by Scheffe’s post hoc test. *** *p* < 0.001. All the cell experiments were repeated independently in triplicate.

We first employed lentiviral packaging to overexpress the four candidate genes in 293T cells. The lentivirus produced from these cells was then used to infect BMSCs using the 293T supernatant. After a 48 h transduction period, RNA was extracted from the infected BMSCs to assess infection efficiency via qRT-PCR. The results confirmed successful infection (Figure 6A).

To assess the impact of gene overexpression on osteogenesis, we performed experiments using lentiviruses containing each of the four genes to infect BMSCs. The osteogenic potential of the infected BMSCs was evaluated using ARS on Day 7 post-infection. A control group, infected with a vector virus, was used for comparison. *BCL6* and *TTPAL* were shown to enhance osteogenic potential, as indicated by increased ARS activity (Figure 6B). In contrast, *PTBP1* and *H2AFZ* appeared to inhibit osteogenesis, as demonstrated by reduced ARS activity (Figure 6C). To rule out the influence of apoptosis, flow cytometry analysis was performed, demonstrating that *PTBP1* and *H2AFZ * inhibit osteogenesis independently of apoptosis (Figure 6D).

The aforementioned findings suggest that *BCL6* and *TTPAL* serve as positive regulators of osteogenic lineage differentiation in BMSCs, while *PTBP1* and *H2AFZ* act as negative regulators.

## 4. Discussion

With the advancement of omics, research on genome-wide dynamics, including transcription factors and epigenomic programming, during pre-osteoblast differentiation has made significant progress [44,45]. In this research article, we leveraged publicly available transcriptomic datasets, including GSE37558 and GSE28205, which provided a foundational framework for examining critical factors that determine MSC differentiation fate, to systematically investigate the dynamic regulation of MSC differentiation during osteogenesis by exploring key cellular factors [33,34]. By integrating transcriptomic sequencing from different induction time points in a single-batch microarray dataset, as well as multiple-batch array sequencing, we identified differentially expressed genes that exhibited continuous and significant changes during MSC osteogenic induction.

To enhance the biological and clinical relevance of these candidate genes, we examined their expression levels in human tissues using the HUMAN PROTEIN ATLAS database [46,47]. High expression in blood and immune-related organs was used as a key criterion for further refining our selection. Through extensive big data analysis, we identified 13 genes with elevated expression in one or more blood and immune system tissues (Table 1).

Among these, four genes—*PTBP1*, *H2AFZ*, *BCL6*, and *TTPAL*—were selected for experimental validation due to their high expression in multiple blood and immune tissues. Our experimental results demonstrated dynamic changes in the expression of these genes during osteogenic induction, as shown in Figure 5A, which aligned with transcriptomic sequencing data. Moreover, the functional roles of these genes were confirmed through biological assays, as depicted in Figure 5B. These results validate our approach of combining transcriptome sequencing with big data mining to identify key targets that dynamically regulate biological functions.

Based on previous studies, our research provides deeper mechanistic insights through the integration of the existing databases and high-throughput transcriptomics to systematically explore key regulatory factors governing MSC osteogenesis [48,49,50,51,52]. This study holds great implications for understanding the molecular factors that influence MSC differentiation fate and could have significant clinical applications [53]. Specifically, the identification of *PTBP1*, *H2AFZ*, *BCL6*, and *TTPAL* as the regulators of osteogenic differentiation presents novel therapeutic targets for conditions such as osteoporosis and bone fracture healing.

The current studies aimed to shed light on the molecular factors influencing MSC differentiation during osteogenesis. While our findings contribute to this complex landscape, recent advances have revealed multiple regulatory mechanisms. Tu W et al. identified that circ_0005753 interacts with *PTBP1*, which enhances *TXNIP* mRNA stability and elevates *TXNIP* expression [54]. The circ_0005753/*PTBP1*/*TXNIP* signaling pathway ultimately suppresses BMSC osteogenic differentiation. Similarly, Fujie et al. elucidated how Bcl6 governs Stat1 expression to regulate Runx2 nuclear translocation, where Bcl6 deletion elevates Stat1 levels, thereby inhibiting BMP2-stimulated Runx2 nuclear translocation—a critical step in bone formation [55]. In contrast, the role of *H2AFZ* remains somewhat ambiguous. Our results indicate *H2AFZ* down-regulation during osteogenic differentiation, contrasting with Diesel et al.’s observation of *H2AFZ* stability throughout hASC osteogenic differentiation over 28 days [56]. This discrepancy highlights a critical knowledge gap in understanding *H2AFZ*’s role in BMSC osteogenesis—particularly significant given BMSCs’ fundamental role in skeletal development and homeostasis [57].

While our analysis of high-throughput transcriptomic data from the GEO database provided valuable insights into the biological processes underlying osteogenic differentiation [58], it is important to acknowledge the limitations of this approach. Single analyses, although informative, may not fully capture the complexity of these processes [59,60]. Future studies should adopt multi-omics approaches, integrating data from mRNAs, regulatory factors, proteins, and metabolites to construct comprehensive gene regulatory networks. This would help elucidate causal relationships between molecules and provide a deeper understanding of the underlying mechanisms [61].

Despite this study focusing on high-throughput sequencing to identify key molecules in MSC osteogenic differentiation, further validation through both in vitro and in vivo experiments is necessary [62,63]. This will help facilitate the translation of our findings into clinical applications [64].

## Figures and Tables

**Table 1:** Studied gene candidates expressed in blood and immune tissues.

Gene Name	Blood and Immune System				Protein Expressed in the Database of the HUMAN PROTEIN ALTAS
	Bone Marrow	Lymph Node	Tonsil	Spleen	
*CXCL12*	High	Low	Low	Low	Bone marrow poietic cells showed strong nuclear positivity.
*PTBP1*	High	High	High	Medium	Most normal tissues displayed strong nuclear positivity.
*PKM2*	Low	High	High	High	Cytoplasmic expression in most tissues, hepatocytes, neurons, and most soft tissues was negative.
*H2AFZ*	High	High	Medium	High	Ubiquitous nuclear expression.
*NUDT1*	Medium	High	High	Medium	Most normal tissues showed moderate to strong cytoplasmic staining.
*ANGPT1*	High	Medium	Medium	Medium	Ubiquitous cytoplasmic expression.
*PPAGR*	Low	Not detected	High	Low	Squamous epithelia, glandular cells in the gastrointestinal tract, gall bladder, urinary bladder, placenta, and epididymis showed moderate to strong nuclear positivity.
*MME*	Medium	Low	Medium	High	B-lymphocytes, myoepithelium, stromal cells, and some glandular epithelia displayed strong cytoplasmic positivity.
*RPS6KA2*	Medium	Low	High	Medium	Most of the normal tissues displayed moderate nuclear and cytoplasmic positivity.
*TTPAL*	Medium	High	High	High	Most normal tissues displayed moderate to strong cytoplasmic staining with a granular pattern.
*BCL6*	High	High	High	Medium	Nuclear expression, mainly in lymphoid tissues.
*CTNNB1*	Low	Not detected	High	Not detected	Membranous expression was observed in most tissues.
*STAT5A*	Medium	High	High	Low	Cytoplasmic and nuclear expression in a few tissues, most abundant in subsets of lymphoid cells.

## Data Availability

Data supporting our study are openly available in public repositories GSE37558 and GSE28205.

## Supplementary Materials

The following supporting information can be downloaded at https://www.mdpi.com/article/10.3390/genes15121568/s1, Table S1: The primer sequences.
