# Supplementary material for: Exploration of Key Regulatory Factors in Mesenchymal Stem Cell Continuous Osteogenic Differentiation via Transcriptomic Analysis
Source: Genes (Basel). 2024 Dec 4;15(12):1568. doi: 10.3390/genes15121568 (PMC11675713; doi:10.3390/genes15121568)
Supplement: Supplementary file 1 [file genes-15-01568-s001.zip › genes-3290372-supplementary.pdf]

genes

Table S1. The primer sequences.

Primer Information	Primer Sequence
Gapdh qPCR Forward Primer	TGGCCTTCCGTGTTCCTAC
Gapdh qPCR Reverse Primer	GAGTTGCTGTTGAAGTCGCA
Alpl qPCR Forward Primer	GGCTGGAGATGGACAAATTCC
Alpl qPCR Reverse Primer	CCGAGTGGTAGTCACAATGCC
Bglap qPCR Forward Primer	CTGACCTCACAGATGCCAAGC
Bglap qPCR Reverse Primer	TGGTCTGATAGCTCGTCACAAG
H2afz qPCR Forward Primer	CCAAGACAAAGGCGGTTTCC
H2afz qPCR Reverse Primer	TTTCAGGTGTCGATGAATACGG
Bcl6 qPCR Forward Primer	CCGGCACGCTAGTGATGTT
Bcl6 qPCR Reverse Primer	GCACTGTCTTATGGGCTCTAAAC
Ttpal qPCR Forward Primer	GGCCTCACTCTCCGAAAATGA
Ttpal qPCR Reverse Primer	CAGGTATGGGTACTCCTTCCG
Ptbp1 qPCR Forward Primer	GCAGGCTGTAAACTCCGTCC
Ptbp1 qPCR Reverse Primer	GGGTCACTGGGTAGAAAAGGTT
